# Potentially Inappropriate Prescribing in Older Primary Care Patients

**DOI:** 10.1371/journal.pone.0095536

**Published:** 2014-04-24

**Authors:** Sandra Vezmar Kovačević, Mika Simišić, Svetlana Stojkov Rudinski, Milica Ćulafić, Katarina Vučićević, Milica Prostran, Branislava Miljković

**Affiliations:** 1 Department of Pharmacokinetics and Clinical Pharmacy, University of Belgrade, Faculty of Pharmacy, Belgrade, Serbia; 2 Pharmacy Subotica, Subotica, Serbia; 3 Department of Pharmacology, Clinical Pharmacology and Toxicology, University of Belgrade, Faculty of Medicine, Belgrade, Serbia; University of Glasgow, United Kingdom

## Abstract

**Objectives:**

The aim of the study was to determine the rate of Potentially Inappropriate Medicines (PIM) and Potential Prescription Omissions (PPO) according to Screening Tool of Older Person's potentially inappropriate Prescriptions/Screening Tool to Alert doctors to the Right Treatment (STOPP/START) criteria.

**Study Design:**

A cross-sectional survey in community pharmacy.

**Method:**

A prospective cross-sectional study was performed, during March-May 2012, in five community pharmacies. Patients aged ≥65 years, who collected one or more prescribed medications, were asked to participate in the study, and an interview was scheduled. Patients were asked to provide their complete medical and biochemical record from their general practitioner.

**Results:**

509 patients, mean age 74.8±6.5 years, 57.4% female, participated in the study. 164 PIM were identified in 139 patients (27.3%). The most common were: long-term use of long-acting benzodiazepines (20.7%), use of non-steroidal antiinflammatory drugs (NSAID) in patients with moderate-severe hypertension (20.1%), use of theophylline as monotherapy for chronic obstructive pulmonary disease (COPD, 15.9%) and use of aspirin without appropriate indication (15.2%). Patients with more than four prescpritions had a higher risk for PIM (OR 2.85, 95% CI 1.97–4.14, p<0.001). There were 439 PPO, identified in 257, (50.5%) patients. Predictors for PPO were older age, presence of diabetes, myocardial infarction, osteoporosis, stroke, COPD and/or angina pectoris.

**Conclusion:**

STOPP/START criteria may be useful in identifying inappropriate prescribing and improving the current prescribing practices. Pharmacists should focus more on patients with more than four medications and/or patients with gout or pain accompanied with arterial hypertension because those patient may be at higher risk of PIM. Additionlly, patients older than 74 years with diabetes, osteoporosis, myocardial infarction, stroke, angina pectoris and/or COPD may have an increased risk of PPO.

## Introduction

Older people have an increased risk of multimorbidity and are more likely to be prescribed several medications concomitantly, which increases the risk of adverse drug events (ADE) [1]. Studies have shown that inappropriate prescribing is common in older hospitalized patients [2], [3] and that it may lead to increased risk of hospitalization [4], [5]. A medication is considered inappropriate if the risk of ADE outweighs the clinical benefit, particularly if safer and/or more effective medications are available for the condition. Also, prescription of a medication at an excessive dose, prolonged duration, prone to drug-drug or drug-disease interactions is inappropriate [6]. Moreover, there is an increasing awareness, among health care providers, that omission of potentially beneficial medications from treatment is inappropriate as well [6].

Several tools are available for detecting inappropriate prescribing. The most commonly used worldwide are the Beers' criteria developped by a panel of experts [7]. However, Beers' criteria have several limitations since drug-drug interactions and potentially inappropriate drug omissions are not considered which may lead to underestimation of potentially inappropriate prescribing. Another tool called STOPP (Screening Tool of Older Person's Prescriptions) and START (Screening Tool to Alert doctors to Right Treatment) has been developed by an Irish group of authors [8]. STOPP criteria consist of 65 indicators which detect Potentially Inappropriate Medicines (PIM) including drug-drug and drug-disease interactions. START criteria comprise 22 indicators of Potential Prescribing Omissions (PPO) in older people. Recent reviews endorse the use of STOPP/START over Beers' criteria in detecting inappropriate prescribing in the elderly [9]–[11].

STOPP/START criteria have shown high prevalences of PIM and PPO in hospitalized and nursing homes patients [2], [3], [12], [13]. However, only few studies have adressed the use of these tools in primary care [14], [15]. Pharmacists, as most accesible members of the health care team in primary care, may play an important role in detecting potentially inappropriate prescribing in the older population. Morever, the role of pharmacists was supported by the good inter-rater reliability between pharmacists and physicians when STOPP/START criteria were applied to the same patient profiles [16], [17].

The aim of the study was to determine the rate of PIM and PPO according to STOPP/START criteria in community pharmacies.

## Methods

### Study setting and population

A prospective cross-sectional study was performed on a group of older patients by five community pharmacists in three regions in Serbia. All five pharmacies were located in urban areas. This study was a part of a larger research project initiated by Academic staff, with the intention to promote research among primary and secondary care pharmacists. Within the project, five community pharmacists were trained and assigned to investigate inappropriate prescribing according to STOPP/START criteria.

All patients aged ≥65 years, who presented in the pharmacy in order to collect one or more prescribed medications, were asked to participate in the study. Patient interview was scheduled upon mutual agreement, and patients were asked to provide their complete medical and biochemical record from the past year. On patient's request, all data were issued by the general practitioner, since pharmacists do not have access to electronic patient records.

### Data collection

Data were collected during a 3-month period, between March and May 2012. An Ethical Committee approval for the research was obtained by the Faculty of Pharmacy, University of Belgrade. Patients were informed about the aims of the study and had to give a written consent.

A questionnaire for demographic, clinical and laboratory data which included age, gender, current diagnoses and medical problems, medical histories, current medications and relevant biochemical data was used. In the event that research pharmacists were uncertain about the diagnosis, interpretation of biochemical data and application of screening tools, they reffered to a senior academic pharmacist and two teacher-practitioner pharmacists at the Faculty of Pharmacy.

Application of STOPP/START criteria to collected data was performed by community pharmacists and reviewed by teacher-practitioners.

### Statistical analysis

All data were collected using Microsoft Excel 2003. Statistical analysis was performed with PASW 18.0 (SPSS Inc., Chicago, IL, USA). Continuous varibles in the text and tables were expressed by mean ± S.D. and categorical data were presented as percentage. Multivariate logistic regression was used to determine independent risk factors that were associated with PIM or PPO. Disease/condition with more than one PIM or PPO, age categorized into groups: 65–74, 75–84 and >85 years, number of prescribed drugs categorized into groups: 1–4, 5–8 and >9 and sex were entered in the logistic regression analysis and a model was built using the stepwise method wich excluded variables at a selection threshold of 0.1. The results of the regression analysis are presented with odds ratios (OR) and their 95% confidence intervals (CI). A probability value of <0.05 was considered to be statistically significant.

## Results

### Baseline characteristics

Out of 547 patients who were asked to participate 509 patients (93%) were included in the study. Their characteristics are shown in Table 1. The mean age was 74.8±6.5 years, 57.4% were female. 2621 medications were prescribed and 54 diagnoses were recorded. 37% of patients had more than 5 medications prescribed.

**Table 1:** Characteristics of the study population.

Population characteristics	Total (n = 509)
Age, mean±S.D., range	74.8±6.5, (65–95)
Sex (female), n, (%)	292, (57.4)
Number of drugs prescribed	2621
Drug prescriptions per patient, mean±S.D., range	5.1±2.2, (1–16)
Number of diseases/conditions	54
Number of diseases/conditions per patient, mean±S.D., range	3.1±1.4, (1–8)
Most frequent diagnoses, n, (%)	
Arterial hypertension	460, (90.3)
Diabetes mellitus	148, (29.1)
Cardiac failure	115, (22.6)
Anxiety	96, (18.9)
Angina pectoris	89, (17.5)
Prostate hyperplasia	72, (14.2)
Depression	53, (10.4)

### Potentially Inappropriate Prescribing

According to STOPP criteria 164 PIM were identified in 139 patients (Table 2). 17 out of 65 STOPP indicators identified inappropriate prescribing in this study. The most common were: long-term use of long-acting benzodiazepines (20.7%), use of non-steroidal antiinflammatory drugs (NSAID) in patients with moderate-severe hypertension (20.1%), use of theophylline as monotherapy for chronic obstructive pulmonary disease (COPD, 15.9%), use of aspirin without appropriate indication (15.2%) and duplication of therapy (10.4%) (Table 3).

**Table 2:** Number of potentially inappropriate prescriptions according to STOPP and START criteria.

Number of potentially inappropriate prescriptions	STOPP	START
	n, (%)	n, (%)
1	118, (23.2)	118, (23.2)
2	18, (3.5)	104, (20.4)
3	2, (0.4)	29, (5.7)
4	1, (0.2)	4, (0.8)
5	0	2, (0.4)
Total	164, (27.3)	439, (50.5)

**Table 3:** Most frequently encountered potentially inappropriate prescriptions according to STOPP and START criteria.

STOPP and START criteria	Total
**STOPP**	
Digoxin >125 mg/day in renal impairment	4
Loop diuretics as first-line monotherapy for hypertension	2
Thiazide diuretics with a history of gout	6
β-blocker in combination with verapamil	1
Aspirin with history of peptic ulcer without gastro-protection	2
Aspirin with no history of vascular symptoms or occlusive event	25
Aspirin to treat dizziness without cerebrovascular disease	1
Long-term use of long-acting benzodiazepines	34
Long-term use of neuroleptics as long-term hypnotics	1
Long-term use of neuroleptics in those with Parkinsonism	1
Theophylline as monotherapy for COPD	26
NSAID with history of peptic ulcer without gastro-protection	1
NSAID with moderate-severe hypertension	33
Warfarin and NSAID together	3
Long-term corticosteroids as monotherapy for rehumathoid arthritis	1
Glibenclamide with Type 2 diabetes mellitus	6
Duplication of therapy	17
**START**	
Warfarin in the presence of chronic atrial fibrilation	14
Aspirin or clopidogrel in patients with vascular disease and sinus rhythm	37
Statin therapy with history of vascular disease	15
ACE inhibitor with chronic heart failure	30
ACE inhibitor following acute myocardial infarction	7
β-blocker with chronic stable angina	43
Regular inhaled β_2_ agonist or anticholinergic agent in asthma or COPD	30
L-dopa in idiopathic Parkinsonism	4
Disease-modifying antirheumatic drug in rheumathoid disease	2
Bisphosphonates in patients taking maintenance corticosteroid therapy	2
Calcium and Vitamin D supplementation in osteoporosis	11
Metformin in Type 2 diabetes mellitus	17
ACE inhibitor in diabetes with nephropathy	2
Antiplatelet therapy in diabetes mellitus with cardiovascular risk	108
Statin therapy in diabetes mellitus with cardiovascular risk	117

ACE, angiotensin converting enzyme; COPD, chronic obstructive pulmonary disease; NSAID, non-steroidal antiinflammatory drug.

72.8% of PIM were associated with four diagnoses: osteoarthritic pain, anxiety, COPD and primary prevention of cardiovascular events.

Using multivariate regression analysis, we identified independent risk factors for PIM in our cohort (Table 4). Patients with more than 4 medications were at higher risk for having PIM (5–8 medications OR 2.56, 95% CI 1.75–3.74, p<0.001 and ≥9 medications OR 7.43, 95% CI 3.20–17.23, p<0.001).

**Table 4:** Factors associated with potentially inappropriate prescribing according to STOPP/START criteria.

Factors	STOPP		
	PIM	No PIM	p-value
*Sex (female)*	84	208	n.s
*Number of medications*			
1–4	35	173	
5–8	89	177	<0.001
≥9	15	10	<0.001
*Age*			
65–74	77	195	
75–84	44	148	n.s.
≥85	18	27	n.s.
*Diagnosis*			
Pain/arterial hypertension	31	10	<0.001
Gout	6	5	<0.001
Anxiety	28	68	n.s
Arterial hypertension	41	419	n.s.
Asthma	2	14	n.s
Depression	9	44	n.s
Diabetes mellitus	6	142	n.s.
COPD	24	23	n.s.
Myocardial infarction	3	33	n.s.
Rheumathoid arthritis	4	11	n.s.
Cardiac failure	5	110	n.s.
Sleep disorder	3	9	n.s.

	START		
	PPO	No PPO	p-value
*Sex (female)*	181	111	n.s.
*Number of medications*			
1–4	103	105	
5–8	135	131	n.s.
≥9	19	16	n.s.
*Age*			
65–74	124	148	
75–84	106	86	0.041
≥85	27	18	0.009
*Diagnosis*			
Diabetes mellitus	135	13	<0.001
Osteoporosis	11	3	<0.001
Myocardial infarction	28	8	<0.001
Stroke	15	5	<0.001
COPD	27	20	<0.001
Angina pectoris	47	42	<0.001
Atrial fibrillation	12	15	n.s
Cardiac failure	29	86	n.s
Parkinsonism	4	7	n.s
Prosthetic heart valve	2	9	n.s
Arterial hypertension	2	458	n.s
Asthma	3	13	n.s
Rheumathoid arthritis	3	12	n.s

COPD, chronic obstructive pulmonary disease; n.s. not significant; PIM, Potentially Inappropriate Medication; PPO, Potential Prescription Omission; START, Screening Tool to Alert doctors to the Right Treatment; STOPP, Screening Tool of Older Person's potentially inappropriate Prescriptions.

A statistically significant higher risk for PIM was identified for pain accompanied by arterial hypertension (OR 31.27, 95% CI 15.01–65.17, p<0.001) and gout (OR 10.26, 95% CI 3.10–34.01, p<0.001). Patients with osteoarthritic pain and arterial hypertension were often prescribed prolonged NSAID treatment (75.6%). Gout was associated with the prescribing of thiazide diuretics (54.5%).

### Potential Prescription Omissions

START identified a total of 439 PPO in 257 (50.5%) patients (Table 2). 15 of the 22 START criteria identified omissions in this study. The cardiovascular and endocrine system accounted for most PPO (88.6%). Lack of antiplatelet therapy and statins in patients with history of coronary, cerebral or peripheral vascular disease or in patients with diabetes mellitus with co-existing major cardiovascular risk factors were the most common omissions. Morover, β-blockers were omitted in the treatment of patients with angina pectoris and patients with COPD were omitted regular inhaled β_2_-agonists or anticholinergics. No omissions were identified under the gastrointestinal system criteria.

Patients older than 74 years were more likely to have a PPO (75–84 years OR 1.47, 95% CI 1.01–2.13, p = 0.041 and ≥85 years OR 1.79, 95% CI 1.19–2.83, p = 0.009).

Following diagnoses were identified as indipendent predictors of prescribing omissions (Table 4): diabetes (OR 69.55, 95% CI 38.55–125.49, p<0.001), myocardial infarction (OR 14.82, 95% CI 6.69–32.86, p<0.001), osteoporosis (OR 14.73, 95% CI 4.08–53.11, p<0.001), stroke (OR 12.19, 95% CI 4.40–33.80, p<0.001), COPD (OR 5.64, 95% CI 3.12–10.20, p<0.001) and/or angina pectoris (OR 3.85, 95% CI 2.49–5.96, p<0.001). Patients with diabetes and increased cardiovascular risk lacked statins in their treatment in 91.2% of cases. Osteoporosis was associated with lack of supplementation with calcium and vitamin D (78.6%) whereas angina pectoris was not treated with β-blockers (47.2%) and COPD patients lacked regular inhaled β_2_ agonists or anticholinergics (57.5%). Moreover, 75% of patients with myocardial infarction or stroke were associated with at least one omission regarding use of aspirin, statin or ACE inhibitor, where indicated.

## Discussion

The results of the study indicate a substantial rate of PIM and PPO in a cohort of older primary care patients in Serbia. The prevalence of PIM among primary care patients in our study was comparable to other reports (14.8–36.0%) [15], [18], [19]. Higher prevalence of PIM was reported in hospitalized patients, patients on admission to hospital (35–79%) [2]–[4], [20]–[22] and in nursing homes (59.8–79%) [12], [13].

We observed not only a difference in prevalence of PIM among reports but also a different pattern of inappropriate prescribing in primary and secondary care patients. Most frequent PIM in our study were associated with five STOPP indicators: long-term use of long-acting benzodiazepines, NSAID use in patients with moderate to severe hypertension, theophylline monotherapy in patients with COPD, aspirin in patients with no history of vascular symptoms and occlusive events and duplication of therapy. The results of Ryan et al. [15] were similar to some extent and reported a high occurrence of proton pump inhibitor (PPI) use for more than 8 weeks (29.4%), long-term use of long-acting benzodiazepines (19.9%), NSAID use in patients with moderate to severe hypertension (11.3%), duplication of therapy (8.4%) and the use of a cardioselective β-blocker in COPD (6.4%), whereas Yayla et al. [18] reported duplication of therapy (64.6%) and aspirin use (18.8%) to be most frequent.

In contrast, reports from hospitalized patients revealed higher prevalence of inappropriate prescribing in patients prone to falls (14.2–15.2%) and use of calcium channel blockers in chronic constipation (4.2–12.3%). The five most common STOPP indicators accounted for only 20.3–38.7% of PIM [2], [3]. Prevalence of PPI use for more than 8 weeks (8.2%), use of aspirin in patients with no history of vascular symptoms and occlusive events (4.9%) and use of long-term long-acting benzodiazepines (2.5–4%) was lower compared to primary care [2], [3].

The START tool, used together with STOPP criteria, enabled a more complete assessment of potential inappropriate prescribing in older people. PPO in our population were more frequent compared to the Irish population (50.5% vs 22.7%) [15]. However, our results were comparable to those in hospitalized patients (34–59.4%) [2], [3], [21], [23] and nursing homes (42.2–74%) [12], [13]. Most prevalent were PPO in patients with cardiovascular diseases, diabetes, osteoporosis and COPD, in all assessed studies [2]–[4], [13]. We found highest prevalence of PPO in patients with diabetes followed by cardiovascular diseases which is in concordance with the results in Taiwanese hospital patients [2]. Other studies reported highest prevalence of PPO in cardiovascular patients (31.5–55.6%) [3], [15].

Number of medications prescribed for PIM and age for PPO were identified as independent risk factors in our study. Similar results were reported by Gallagher et al. [3] while other reports identified number of medication and age as independent risk factors for PIM but not for PPO [2], [15].

Our results revealed the presence of a disease/condition as an independent risk factor for PIM/PPO. Pain in patients with moderate to severe hypertension and/or gout were predictors for PIM, whereas the presence of diabetes mellitus, osteoporosis, myocardial infarction, stroke, COPD and angina pectoris were independent risk factors for PPO. There is some discrepancy between most frequent STOPP indicators and the diseases which predispose to PIM. This may be explained by different indications for the use of long-acting benzodiazepines which were encountered among our patients, the inability to associate therapy duplication with a specific disease and the high prevalence of hypertension associated with primary prevention aspirin use. In opposite, the diseases/conditions predisposing to PPO were in accordance with most frequent START indicators.

Several studies have shown a beneficial impact of community pharmacists in improving older patients' health care outcomes [24]–[27]. The use of STOPP/START criteria in improving health care outcomes in primary care, remains to be established. However, recent findings in secondary care may be promising [28]–[30]. It has been argued that primary care pharmacists may overestimate the rate of PIM and underestimate the rate of PPO when using STOPP and START criteria due to lack of clinical information [14]. In some cases, what is considered inappropriate according to STOPP/START may be appropriate for the individual patient for many reasons and this is difficult to assess without communication with the prescriber. Our pharmacists reviewed patient's medical and biochemical data based on the general practitioner's report. The similar results of PPO in our study compared to hospitalized patients may indicate the robustness of START criteria in primary and secondary care. Nevertheless, we observed differences in PIM between primary and secondary care patients. The reasons for the difference in the number and prevalence of different PIM are probably multifactorial. Hospitalized patients are generally sicker and frailer than primary care patients. Older age, more medicines prescribed, more comorbidities and a higher severity of illness could have accounted for the number and diversity of PIM.

There were some limitations to our study. Number of patients for data collection as well as exclusion of patients who did not claim prescriptions personally, limits the generalizability of findings. Furthermore, clinical information for assessing inappropriate prescribing and omissions may have been incomplete since their was no close communication with prescribers. Nevertheless, this study showed that community pharmacists were able to identify many cases of inappropriate prescribing using easily applicable screening tools such as STOPP/START criteria.

## Conclusion

27.3% of patients with PIM and 50.5% with PPO were observed in our study indicating that STOPP/START criteria may be useful in identifying inappropriate prescribing and improving the current prescribing practices. Pharmacists should focus more on patients with more than four medications and/or patients with gout or pain accompanied with arterial hypertension because those patient may be at higher risk of PIM. Additionlly, patients older than 74 years with diabetes, osteoporosis, myocardial infarction, stroke, angina pectoris and/or COPD may have an increased risk of PPO.
